# Dataset for the validation and use of DiameterJ an open source nanofiber diameter measurement tool

**DOI:** 10.1016/j.dib.2015.07.012

**Published:** 2015-07-29

**Authors:** Nathan A. Hotaling, Kapil Bharti, Haydn Kriel, Carl G. Simon

**Affiliations:** a Biosystems & Biomaterials Division, National Institute of Standards & Technology, Gaithersburg, MD, 20899; b Unit on Ocular and Stem Cell Translational Research, National Eye Institute, National Institutes of Health, Bethesda, MD 20892; c The Stellenbosch Nanofiber Company (Pty) Ltd, Cape Town, South Africa

## Abstract

DiameterJ is an open source image analysis plugin for ImageJ. DiameterJ produces ten files for every image that it analyzes. These files include the images that were analyzed, the data to create histograms of fiber radius, pore size, fiber orientation, and summary statistics, as well as images to check the output of DiameterJ. DiameterJ was validated with 130 *in silico*-derived, digital, synthetic images and 24 scanning electron microscope (SEM) images of steel wire samples with a known diameter distribution. Once validated, DiameterJ was used to analyze SEM images of electrospun polymeric nanofibers, including a comparison of different segmentation algorithms. In this article, all digital synthetic images, SEM images, and their segmentations are included. Additionally, DiameterJ’s raw output files, and processed data is included for the reader. The data provided herein was used to generate the figures in *DiameterJ: A Validated Open Source Nanofiber Diameter Measurement Tool*[1], where more discussion can be found.

**Specifications Table**

**Table:** 

Subject area	Bioengineering and Materials Science
More specific subject area	Tissue Engineering and materials characterization
Type of data	Tables, images, graphs/figures, text files, picture document files
How data was acquired	Digitally generated images made using Inkscape graphics package. Scanning electron microscope images (SEM, Hitachi S4700 SEM, 5 kV, 10 mA, ≈13 mm working distance).
Data format	Raw, filtered, and analyzed
Experimental factors	All .svg files generated by Inkscape were converted to .tif files using ImageJ. All SEM images were first segmented using default algorithms in ImageJ as per the methods discussed in [1].
Experimental features	DiameterJ was written as a plugin for ImageJ and validated using images generated in Inkscape graphics package. Images were analyzed by other software packages and methods and results were compared with DiameterJ. Next, DiameterJ was used to assess SEM images of steel wires with a known diameter distribution. Other available software/methods were also used to analyze the steel wire images and results were compared to DiameterJ. SEM images of electrospun polymeric nanofiber were also analyzed with DiameterJ and other available software/methods, and results were compared. Finally, different segmentation algorithms were tested with DiameterJ to assess its robustness when analyzing images of different segmentation quality.
Data source location	Gaithersburg Maryland, United States. 39 °08′11.2“N 77 °13′09.3“W
Data accessibility	Data is Zipped within this article. Also the source code for DiameterJ can be found at https://github.com/NHotaling/DiameterJ

**Value of the data**

**Table:** 

•	The images provided here can be analyzed by DiameterJ users to establish comparability with our results and demonstrate that they are using the program appropriately.
•	The images can also be used by nanofiber measurement software developers to use when validating their software. This has value because expensive electron microscopes are needed to generate reference images and thus this dataset expands the potential pool of software developers to those who cannot afford or do not have access to electron microscopes.
•	The digital synthetic images and steel wire images can be used by others to validate their fiber dia. analysis methods. This will have value by improving the comparability between the output of other researchers’ nanofiber diameter analysis tools by providing a common benchmark.
•	The data that support the validation of the DiameterJ are provided. The value in these data is that others can see the strategy employed to validate DiameterJ in order to use or improve this strategy when validating other image analysis algorithms. Strategies to identify a ground truth are not easy to establish and require careful consideration.

## 1 Data

Three sets of images used to validate and test DiameterJ are provided: synthetic digital images with known fiber diameters, SEMs of steel wires of known diameters and SEMs of electrospun polymeric nanofibers of unknown diameter.

### 1.1 Experimental Design

DiameterJ is a user friendly ImageJ/FIJI plugin that can analyze SEM micrographs of nanofibers to determine nanofiber diameter on a desktop computer within 60 s [1].

We generated sets of digital synthetic “calibration” images using software in which white lines/fibers, with a defined diameter, were drawn on a black background. Images were created that had lines that were in both ordered and disordered orientations. Additionally, images were created with multiple different fiber diameters in a single image. The DiameterJ code was modified until it produced low error on these “calibration” images. Next, SEM images of micro-scale stainless steel wire of known diameter distribution were analyzed to demonstrate that DiameterJ was effective for analyzing SEM images. The DiameterJ results were compared to other available algorithms and to Human measures of the images. After determining that DiameterJ was able to analyze these images with relatively low error, as compared to other available software and human measurement, DiameterJ was tested on segmented images of poly(lactic-co-glycolic acid) polymer nanofibers, of unknown diameter. Next, the results of DiameterJ’s analysis of the polymer nanofiber images was compared to human measures and commercial software for these images. Finally, the effect that different segmentation algorithms of the same image had on DiameterJ’s outputs was analyzed and compared to determine the robustness of the algorithm to segmentation algorithm.

## 2 Methods

### 2.1 Calibration Image Generation

For images with ordered straight lines, a black box with a 1280×960 pixel (px) resolution was created and ordered white lines (fibers) were drawn using the Beziers drawing tool with a variety of defined line diameters. The angle of white line orientation for the ordered straight line calibration images was specified in Inkscape. In addition, disordered curved lines that more closely mimic electrospun nanofiber morphologies were drawn with the Freehand tool in Inkscape. Calibration image line diameters were manually confirmed by hand by counting pixels on screen at 25 locations per image to confirm line diameters.

Calibration images were created in sets of three. For example a set of three images containing ordered straight lines with a 25 px diameter was created where the images were very similar but the exact arrangement and angles of the lines was varied. This set of three images was used to determine the ability of the programs to measure the diameter of 25 px lines. Five types of image sets were created: sets with straight lines of a single dia. (Ordered-1D); sets of disordered, curved lines of a single dia. (Disordered-1D); sets of straight lines of 3 dia. (Ordered-3D); sets of disordered, curved lines of three dia. (Disordered-3D); and sets of disordered, curved lines of 2, 3, 4 or 6 dia. (Multi-Dia.). For Ordered-3D and Disordered-3D, each image in the sets of three had the same overall average line dia. For Multi-Dia., each image in the sets of three had a different overall average line dia. To limit bias in fiber diameter selection or frequency, Excel’s random number generator was used for the selection of line diameter and its frequency within the Ordered-3D, Disordered-3D and Multi-Dia. image sets The images and their analysis as well as the excel file containing the algorithm for random fiber diameter generation can be seen in Data Folder 1 and Data Folder 2.

### 2.2 Segmentation and Image Processing of SEM Images

Image segmentation was performed via eight techniques: (i) global thresholding methods developed by Otsu[2], (ii) Huang[3], or (iii) Minimum Error[4]; (iv, v & vi) local adaptive thresholding using the same segmentation algorithms as for global, but with a local window of the mean fiber diameter (MFD) plus 10%; (vii) machine learning techniques[5] and (viii) edge detection methods[6]. For the machine learning technique, training features were set to Gaussian blur, hessian, membrane projections, Sobel filters, difference of Gaussians, grey variance, Laplacian, and structure of classes with a membrane patch size of 19 px with minimum/maximum sigma values of 1 and 16, and membrane thickness of 1 px. For machine learning, two images were used to select class 1 (fiber) and class 2 (background) features to create a classifier with an average of 20 features being selected for each class. A unique classifier was created for both the wire and PLGA nanofiber SEM micrographs. A macro was written to run all segmentation methods on every image. The segmentations were visually compared to the original SEM micrographs and the segmentation that most closely resembled the original SEM was chosen for analysis.

After segmentation, images were smoothed according to D’Amore et al. [7] Briefly, successive rounds of noise removal (via ImageJ’s despeckle command) were performed until no change in the image was found. A sequence of erosion (through ImageJ’s Erode command), dilation (through ImageJ’s dilate command), and an additional erosion operation served to refine the image, highlighting fiber edges and eliminating isolated pixel areas [8]. The described morphological procedures were performed to improve the precision of the centerline determinations as per the method developed by Lam et al. [9]. Three representative images can be seen in Figure S4 of reference [1], the before and after of a global (Otsu), local (Local Otsu), and machine learning thresholding steps.

### 2.3 Image Analysis

*BoneJ:* BoneJ [10] is a plugin for ImageJ with an algorithm called Thickness that is a modification of Dougherty et al. local thickness plugin [11] and included for comparison to DiameterJ. BoneJ’s Thickness defines the structure “thickness” at a point as the diameter of the largest circle (2D) that fits within the structure and which contains the point. For BoneJ analysis of calibration images or segmented SEM micrographs, the images were opened in ImageJ and color-inverted (BoneJ analyzes black objects). The Thickness algorithm within BoneJ was selected and “Thickness” was checked in the window. BoneJ outputs a line diameter and a standard deviation but does not provide a distribution of fiber diameters.

*FibraQuant:* FibraQuant (NanoScaffold Technologies, LLC) is a commercially licensed algorithm and included for comparison to DiameterJ. FibraQuant outputs a histogram of fiber diameters and pore metrics as well as fiber orientation. For all SEM images, Fibraquant histogram data was analyzed and peak-fit (Gauss) in an identical manner to that described for DiameterJ Histogram.

*Single, Ordered-3D and Disordered-3D Calibration Images:* The percent error of all programs and methods was calculated as follows: $\%\;error=(|D_{Real}-D_{Calc}|/D_{Real})$. For single dia. calibration images, D_Real_ was the line dia. for the given calibration image. For DiameterJ Super Pixel and BoneJ, D_Calc_ was the single value output from the algorithm. For DiameterJ Histogram calculation, D_Calc_ was the average of the line diameters determined at each pixel. For Ordered-3D and Disordered-3D images, D_Real_ was the number average line diameter for all lines in the image and D_Calc_ was the same as used for single dia. images.

*Peak-Fitting:* Peak-fitting was used to determine the fiber diameters in DiameterJ Histogram data, cumulative human measures and FibraQuant’s histogram data. Histograms for Multi-Dia. calibration images and SEM images were analyzed using Igor Pro (WaveMetrics) and fit with a Gaussian curve. Standard deviations of fiber diameter from DiameterJ Histogram were determined as follows:

$$\;St.\;Dev.=\sqrt{\frac{\sum^{\;}{\left(\left(\frac{W}{2⁎\sqrt{2⁎\;\ln\;2}}\right)\times \sqrt{N}\right)}^{2}}{T}},$$

where *W* is Gaussian peak full width half maximum (FWHM), *N* is number of peaks and *T* is total number of images (6 images).

Three Multi-Dia. images of 1, 2, 4 or 6 line diameters were analyzed by the algorithm and the average of each images’ peak error was taken while combining the standard deviations from each image as discussed in the Statistical Analysis section below. Percent error was calculated by taking the absolute value of the known line diameter at the peak minus the modal peak location value divided by the known diameter value. The average of each images’ peak percent error was taken while combining peak standard deviations as indicated above.

*Multi-Dia. Calibration Images:* The percent error was calculated as follows:

$$\%\;error=\frac{\sum^{\;}\frac{\left|D_{Real}-D_{Calc}\right|}{D_{Real}}}{N}.$$

The absolute error (in pixels) was calculated as follows:

$$Abs.\;error=\frac{\sum^{\;}|D_{Real}-D_{Calc}|}{N}.$$

For both percent error and absolute error, N was the number of line diameters in the calibration image set ( 1, 2, 4 or 6), D_Real_ was the line diameter for the different lines in the given calibration image and D_Calc_ was the peak of the Gaussian fit to each mode of the line diameter histogram. Absolute error is provided to show that the errors in DiameterJ’s Histogram algorithm were consistent and low across all multi-diameter images. Absolute error is provided to enable direct comparison of the DiameterJ Histogram results for fibers of different diameters.

*Reference Wire Images:* The percent error was calculated as follows:

$$\%\;error=\frac{\sum^{\;}\frac{\left|D_{Real}-D_{Calc}\right|}{D_{Real}}}{N},$$

where N was the number of wire diameters in the image set (1 or 3), D_Real_ was the wire diameters determined by optical microscopy and D_Calc_ was the peak of the Gaussian fit to each mode of the line diameter histogram.

## 3 Data

### 3.1 Data Folder 1

**Data Folder 1** contains all of the files used to generate Figure 2 in reference [1]. When the file is unzipped six excel files and four folders can be found. In the folder labeled “Tiffs” you can find every digital synthetic image analyzed to produce Figure 2 in reference [1]. Images are named for ease of reference. To summarize, images with only single diameters, i.e. all files labeled “003-1.tif - 250-3.tif” and “Dis_003-1.tif - Dis_250-3.tif” are numbered in sequential order. The first number in the name represents the fiber diameter in these images. The second number represents the index of the image with that particular fiber diameter. The images with “Dis_” as a prefix have disordered fiber orientations. Similarly, images with three different fiber diameters, i.e. all files labeled “Ave 10-1.tif – Ave 90-3.tif” and “Dis_Ave 20-1.tif - Dis_Ave 91-3.tif” are numbered in sequential order. The first number in the name represents the global average fiber diameter in these images. The second number represents the index of the image with that particular mean fiber diameter. The images with “Dis_” as a prefix have disordered fiber orientations. In the Tiffs folder a pdf file is also available (called “All Digital Synthetic Images.pdf”) that shows a thumbnail of every digital synthetic tiff file analyzed in this report.

In the excel file titled “Figure 1 – Image Name Descriptions.xlsx” the names of all of the digital synthetic images that were analyzed in Figure 2 of Reference [1] are explained using the description above. Additionally, images with three diameters are further explained with this file by breaking down, for each image, the fiber diameter, the frequency of each fiber diameter occurring in the image, and for the ordered images the length of the line with each diameter. A column showing the average fiber diameter is also shown for each image.

The file labeled “Figure 1A- Overall Summary – Ordered-1 Diam-DSI.xlsx” contains a copy of the raw summary data exported by DiameterJ when analyzing ordered single diameter images. Additionally, rows for the output of BoneJ can be seen below the standard output of DiameterJ. Below the raw data is the mean, standard deviation and percent error at each fiber diameter for each set of three images. Below these summary statistics a condensed table of only the percent error for each fiber analysis technique at each fiber diameter can be seen. Finally, the percent error data used to create Figure 2A in reference [1] can be found along with the subsequent plot of this data.

The format of “Figure 1A- Overall Summary – Ordered- 1Diam-DSI.xlsx” is followed exactly for files: “Figure 1B - Overall Summary - Disordered-1 Diam-DSI.xlsx”, “Figure 1C - Overall Summary - Ordered-3 Diam-DSI.xlsx”, and “Figure 1D - Overall Summary - Disordered-3 Diam-DSI.xlsx”.

The file labeled “Random Diameter Generator.xlsx” contains a copy of the random number generation algorithms used to select the diameters found in Images “Ave 10-1.tif – Ave 90-3.tif” and “Dis_Ave 20-1.tif - Dis_Ave 91-3.tif”. Column A in this sheet contains randomly generated fiber Diameter Frequencies between 1 and 25 depending on how high the general average of the fiber diameters needs to be. Column B is a non-zero form of column 1 where any 0 values in column 1 are changed to a value of 1. Column C contains randomly generated diameters of fibers. Column E contains the summary statistic “Frequency⁎Diameter”. The rest of the spreadsheet is devoted to determining the mean diameter of the fibers chosen along with random number seed generators. To update the sheet and generate new diameters, users need only to press and hold the control key while simultaneously pressing the “R” key to “refresh” the document.

The other three folders included in this zip file contain the raw output of DiameterJ. These files are included so that the reader can delve into where the numbers came from in the files discussed above. An in-depth description of each file and its output can be found on the ImageJ repository http://imagej.net/DiameterJ.

### 3.2 Data Folder 2

**Data Folder 2** contains all of the files used to generate Figure 3 in reference [1]. When the file is unzipped four excel files and five folders can be found. In the folder labeled “Tiffs” you can find every digital synthetic image analyzed to produce Figure 3 in reference [1]. Image names for these files indicate how many fiber diameters are found in the image. For instance images with M02 in their name have fibers with two diameters while images with M06 in their name have fibers with six diameters. To provide more detail, in the excel file titled “Figure 2 – Image Name Descriptions.xlsx” the composition of each set of images with multiple diameter are further explained by breaking down, for each image, the fiber diameter and the frequency of each fiber diameter occurring in the image. Additionally, a column showing the average fiber diameter is shown for each image.

The file labeled “Figure 2B - Example Histograms - Multi Mode_DSI.xlsx” contains the diameter histograms generated by DiameterJ for each of the images discussed in “Figure 2 – Image Name Descriptions.xlsx”. All diameters are in pixels as no units are assigned for digital synthetic images. Additionally, a plot of the fiber diameter versus its frequency of occurrence can be seen for each of the images in this file. Several of these histograms can be seen in reference [1] Figure 3B.

The file labeled “Figure 2C - Gaussian Peak fit of 2D Multi-Modal File.xlsx” shows the Gaussian peak fit of the diameter histogram generated by DiameterJ of file M02D_3. The histogram has two peaks and for each peak the Gaussian curves which have been fit to them have their amplitude, center, and width shown in the excel file (defined by Igor Pro (Wavemetrics)). The other columns of data in this file show each Gaussian fits’ value at each pixel value given the parameters listed for their amplitude, center, and width. Finally, the raw histogram data for image M02D_3 is shown and a graph showing the overlay of the two Gaussian curves on the raw data can be seen. The graph shown in this file is the same graph seen in reference [1] Figure 3C.

The file labeled “Figure 2D & E - Overall Summary - Multi Mode_DSI.xlsx” shows the data needed to generate Figure 3D and E of reference [1]. In row 1 of this file the name of each image analyzed for this analysis is found. Below each image name are the diameters found from the Gaussian peak fit of their diameter Histogram generated by DiameterJ. Images that contain multiple fiber diameters have multiple values listed under them, i.e. images that had six peaks in their histogram have six values listed under them. The next set of rows below these values is the true line diameter in these images. Below the true fiber diameters are both the percent and absolute error of the Gaussian peak fit as compared to the real value. Finally, the mean and standard deviation of for each group of diameters (groups were defined as images with the same number of different diameters in them) was calculated for both the percent error and absolute error. At the bottom of the document the summary of the means and standard deviations for both the percent and absolute error can be found as well as the graphs of these values for each group of diameters.

A folder called “Peak Fits” is also located in the Data Folder 2 zip file. In this folder there are the peak fit parameters generated by Igor Pro for each of the files. The baseline for the fit, the peak location, height, area, and full width half max (FWHM) are given for each peak that was fit for each image. The other three folders included in this zip file contain the raw output of DiameterJ as discussed above.

### 3.3 Data Folder 3

**Data Folder 3** contains all of the files used to generate Figure 4 in reference [1]. When the file is unzipped four excel files and six folders can be found. In the folder labeled “Tiffs” you can find every SEM image of wires with known diameter distributions that were analyzed to produce Figure 4 in reference [1]. Additionally, the segmented and processed images that were analyzed by DiameterJ to produce the data can also be found in this folder. Image names for these files indicate the wire gauge imaged, and for the files with 03 G in front, these images were of wires with three different gauges. For instance images with 48 g in their name were of wires that were 48 gauge in diameter. To provide more detail, in the excel file titled “Figure 3 – Image Name Descriptions.xlsx” what the gauge of the wire was and what the subscripts mean have been defined for each image name. Files with a “_BW” post script are the segmented files.

The file labeled “Figure 3D - Wire Histogram Summary.xlsx” shows the diameter histogram generated by DiameterJ for each image as well as the cumulative histogram of all of the images of a particular gauge combined into a single histogram. The histograms were originally in pixel units so a conversion column for each set of images has also been added to show the transformation of pixel units to micrometers. The pixel to distance transformation factor can be seen in columns AO to AR. The cumulative histogram for each individual gauge and the mixed gauge images has also been graphed to the right of the data. These histograms can be seen in Figure 4D of reference [1].

The file labeled “Figure 3E.xlsx” shows the calculated wire diameter for the Super Pixel, Histogram, BoneJ, Human, and PrC (proprietary code) algorithms for each of the SEM images with only one gauge of wire in them. The mean and standard deviation of six images for each gauge can also be seen in this sheet. From the means the percent error was also found as was the standard deviation of the error for each algorithm. The mean percent error as well as the standard deviation of the error is shown in a bar graph in this file. This graph is explained in greater detail in Figure 4E of reference [1].

The file labeled “Figure 3F.xlsx” shows the calculated mean wire diameters for the Super Pixel, Histogram, BoneJ, Human, and PrC algorithms for the SEM images with three gauges of wire in each image. From the means the percent error was also found as was the standard deviation of the error for each algorithm. The mean percent error as well as the standard deviation of the error is shown in a bar graph in this file. This graph is explained in greater detail in Figure 4F of reference [1].

Other folders in the “Data Folder 3.zip” file are composed identically to those in Data Folder 2. There is one additional folder in Data file 3.zip that was not found in Data Folder 2. This folder is named “Human Data” and contains the raw human measures of wire diameter for each of the two humans who were averaged together to get the human data described above. The human measures file contains each image name, the 25 measures each human performed on the image and the mean and standard deviation of the diameter measures for each image and person. Additionally, in the “Peak Fitting” Folder four sheets have been added that correspond to the output produced by the proprietary code, PrC. These files are labeled “Prc_48_ga_reported_all_samples.xls, Prc_50g_reported_all_samples.xls, Prc_53_g_reported_all_samples.xls, and Prc_mixed_gauge_reported_all_samples.xls”. Each of these excel files contains 7 sheets. The first is a summary sheet describing the average, standard deviation, and median of fiber diameter, the average fiber orientation, the area covered by fibers the measurement resolution, the number of measures for each image, and the percent of the image that was analyzed. The next six sheets are the individual analysis of each image that the PrC analyzed. Each sheet contains the image mean fiber diameter, standard deviation, median fiber diameter, average orientation, and percent area covered by fibers. Additionally, a histogram of diameter values is given as well as an image where all fiber measurements that the PrC made are shown. Finally, a list of each individual diameter measurement as well as the fiber orientation at that measurement is given. This format is consistent for all of the PrC files.

### 3.4 Data Folder 4

**Data Folder 4** contains all of the files used to generate Figure 5 in reference [1]. When the file is unzipped four excel files and five folders can be found. In the folder labeled “Tiffs” you can find every SEM image of PLGA nanofibers with both monodispersed (Mono) and bimodal (Bimodal) fiber diameter distributions that were analyzed to produce Figure 5 in reference [1]. Additionally, the segmented and processed images that were analyzed by DiameterJ to produce the data can also be found in this folder. Image names for these files indicate whether the image contained Mono or Bimodal fibers. Files with a “_BW” post script are the segmented files.

The file labeled “Figure 4C and D - Histogram Algorithm - Mono and Bimodal.xlsx” contains two sheets one for the analysis of Mono dispersed fibers, labeled “Mono” and the other for the analysis of Bimodal fibers, labeled “Bimodal”. Each sheet shows the histogram algorithm’s output for each of the images analyzed and a cumulative histogram summing the frequency of occurrence of all of the measures from each file. The values for the Gaussian peak fit of each curve can also be found in each sheet as well as a graph of the overlay of the Gaussian peak fit on the cumulative histogram of the data. File “Figure 4C and D - Human 1&2 - Mono and Bimodal.xlsx” has a similar format except that instead of showing the histogram from each measure the cumulative frequency of Human 1 and Human 2 across all images is shown. The cumulative histograms from each of these sheets, from both files, can be seen in Figure 5C and D in reference [1].

The file labeled “Figure 4E - Overall Summary_Mono.xlsx” contains the summary data for each monodispersed nanofiber image directly from DiameterJ as well as output from BoneJ and the mean and standard deviation of the fiber diameter as calculated by PrC and Human methods. The human, PrC, and Histogram measures were derived from the peak fits of the cumulative histograms for each analysis method. More discussion of these peak fits can be found below. The file also contains the pixel to distance transformation for the pixel data output by DiameterJ for each image analyzed. Finally, the graph of each of the mean fiber diameters as well as their standard deviations is shown for each algorithm/method. This graph is explained in greater detail in Figure 5E of reference [1].

The file labeled “Figure 4F - Overall Summary_Bimodal.xlsx” contains the summary data for all of the bimodal nanofibers images. The mean and standard deviation obtained from the peak fits of each of the algorithms’ diameter histograms as calculated for PrC, Human, and DiameterJ Histogram methods can be seen in this file as well as the bar graph showing these means and standard deviations for each algorithm. This graph is explained in greater detail in Figure 5F of reference [1].

Other folders in the “Data Folder 4.zip” file are composed identically to those in Data Folder 3. The exception is for the “Peak Fitting” folder. In this folder, instead of output text files from Igor Pro, peak fit data from a different peak fitting software, Fityk, are found with the analysis of the peak fits for the Mono and Biomodal cumulative histograms for the Histogram algorithm, Human 1 and Human 2, and PrC. The Gaussian peak center, height, area under curve, and FWHM are found in each of the following files “Histogram Algorithm - Bimodal Peak Fit.txt, Histogram Algorithm - Mono Peak Fit.txt, Human 1&2 - Bimodal Peak Fit.txt, Human 1&2 - Mono Peak Fit.txt, PrC - Bimodal Peak Fit.txt, and PrC - Mono Peak Fit.txt” where the initial word(s) indicate which algorithms’ data that the peak fit was performed on. In files named “X_ - Mono and Bimodal.xlsx”, where X indicates the algorithm used, there are three sheets. For all three of these files the sheet labeled “Mono” is the analysis of Monodispersed fiber images, “Bimodal” is the analysis of Bimodal fiber images, and “Raw Data” which is the raw unprocessed data from each algorithm. On the Mono and Bimodal sheets a column for diameter, the frequency of occurrence at that diameter, and the equation of the Gaussian fit of that histogram can be seen as well as a graph of the cumulative histogram both with and without the Gaussian Curve fit. For both Prc and Human analysis the Raw Data sheet contains all of the raw measures for each of the images taken. For the Histogram Algorithm the Raw Data sheet contains the histograms of diameter distribution for each of the images taken and their cumulative frequency. Finally, the total data output by PrC can be found in the files “PrC - Bimodal - All_samples_Raw Data.xls and PrC - Mono - All_samples_Raw Data.xls”. The layout of these files is identical to that which was described above for PrC files.

### 3.5 Data Folder 5

**Data Folder 5** contains all of the files used to generate Figure 6 in reference [1]. When the file is unzipped three excel files and five folders can be found. In the folder labeled “Tiffs” you can find an image of 53 gauge wire along with the output from four different segmentation algorithms on this image: Global Minimum Error (Global Min Error), Global Otsu, Local Otsu, and Machine Learning.

The file labeled “Figure 5B – Diameter Histogram.xlsx” contains the frequency of occurrence of radius and diameter measures, as produced by DiameterJ’s Histogram algorithm, for each segmentation algorithms’ output image. The pixel to micrometer conversion for this image is also present in this file as well as graphs of the histograms for each segmentation algorithms’ output image.

The file labeled “Figure 5C – Orientation Histogram.xlsx” contains the frequency of occurrence of a given fiber orientation as defined by the OrientationJ plugin. To determine fiber orientation, a centerline was drawn on all fibers using the axial thinning technique built into ImageJ. The centerline was enlarged by 2 px (using the Enlarge command in ImageJ/FIJI) to ensure accurate measure of the line. The Fourier gradient was used with a Gaussian window equal in size to a 7x7 pixel box. The subsequent frequency histogram of fiber orientation was then found for each of the segmented images and can be seen in this file. The maximum frequency of orientation can be seen next to the orientation frequency values for each image as well as the total sum of counts. The angle where the maximum frequency of fibers occurred was identified and then a table summing the frequency of fiber distribution as they symmetrically diverged from this maximum orientation angle is then shown in the file. Next to these summing columns the reader can find each successive summed angle divided by the total sum of measures, in the form of a percent. The summed angle at which 50 percent of the total area under the orientation histogram has occurred was then highlighted in green to indicate the angle which should be plugged into the normalized orientation index (NOI) equation. The NOI metric was found in the literature to define the orientation of a scaffold,[7] the NOI was calculated as follows: NOI=(90-x)/90×100, where x is the number of degrees you must expand from the most prevalent orientation angle in both directions encompass 50% of the total orientations. In this way, an NOI close to 0% or 100% indicates highly oriented fibers while an NOI near 50% indicates random orientation. The NOI value for each segmentation image can be found below the summed angle percent columns. Finally, graphs of the orientation for each segmentation algorithm are shown in the file to the right of all data columns. These graphs are explained in greater detail in Figure 6C of reference [1].

The file labeled “Figure 5D – Summary Table.xlsx” contains select output from the summary output of DiameterJ (Mean mesh hole size, Porosity, Intersection density, and Characteristic Fiber Length) for each segmentation algorithms’ image as well as the summary output of the Gaussian peak fits of the diameter histograms, discussed below, and the NOI for each image as discussed above.

Other folders in the “Data Folder 5.zip” file are composed identically to those in Data Folder 4. The exception is for the “Peak Fitting” folder. In this folder the Fityk output text files are present along with.csv files which contain the diameter histogram, generated by DiameterJ, of each segmentation algorithms’ image.

### 3.6 Data Folder 6

**Data Folder 6** contains all of the files used to analyze all of the high resolution images mentioned in the supplemental discussion of reference [1]. When the file is unzipped one excel file and four folders can be found. In the folder labeled “Tiffs” you can find digital synthetic images. Each file’s name is descriptive of the average fiber diameter of all the fibers in that image. For example “3D_Ave 35–2.tif” has fibers with three different diameters, the average of these fibers is 35 pixels and this is the 2^nd^ such file with this average (as indicated by the 2 post script at the end of the image name). The other three folders included in this zip file contain the raw output of DiameterJ. These files are included so that the reader can delve into where the numbers came from in the files discussed above.

In the excel file titled “Figure 6 – Image Name Descriptions.xlsx” the names of all of the digital synthetic images are explained by breaking down, for each image, the fiber diameter and the frequency of each fiber diameter occurring in the image. Additionally, a column showing the average fiber diameter is shown for each image.
