# Supplementary material for: Dataset for the validation and use of DiameterJ an open source nanofiber diameter measurement tool
Source: Data Brief. 2015 Jul 29;5:13–22. doi: 10.1016/j.dib.2015.07.012 (PMC4556745; doi:10.1016/j.dib.2015.07.012)
Supplement: Supplementary file 1 — Supplementary data [file mmc1.zip › Data Folder 1.zip!/Data Folder 1/Tiffs/001_All Digital Synthetic Images.pdf]

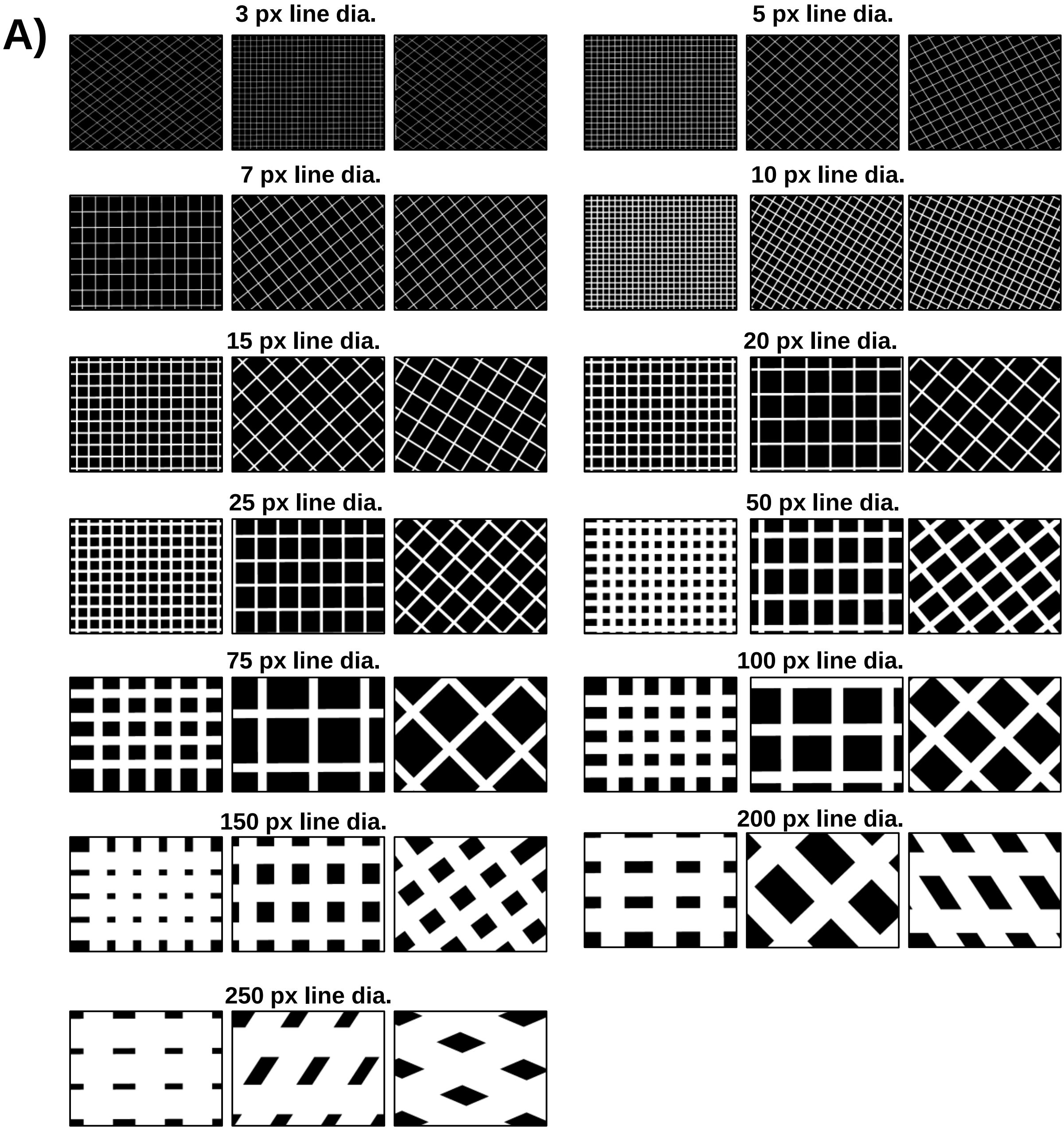

Figure S1A: Calibration Images Ordered, 1 Diameter (Ordered-1D)

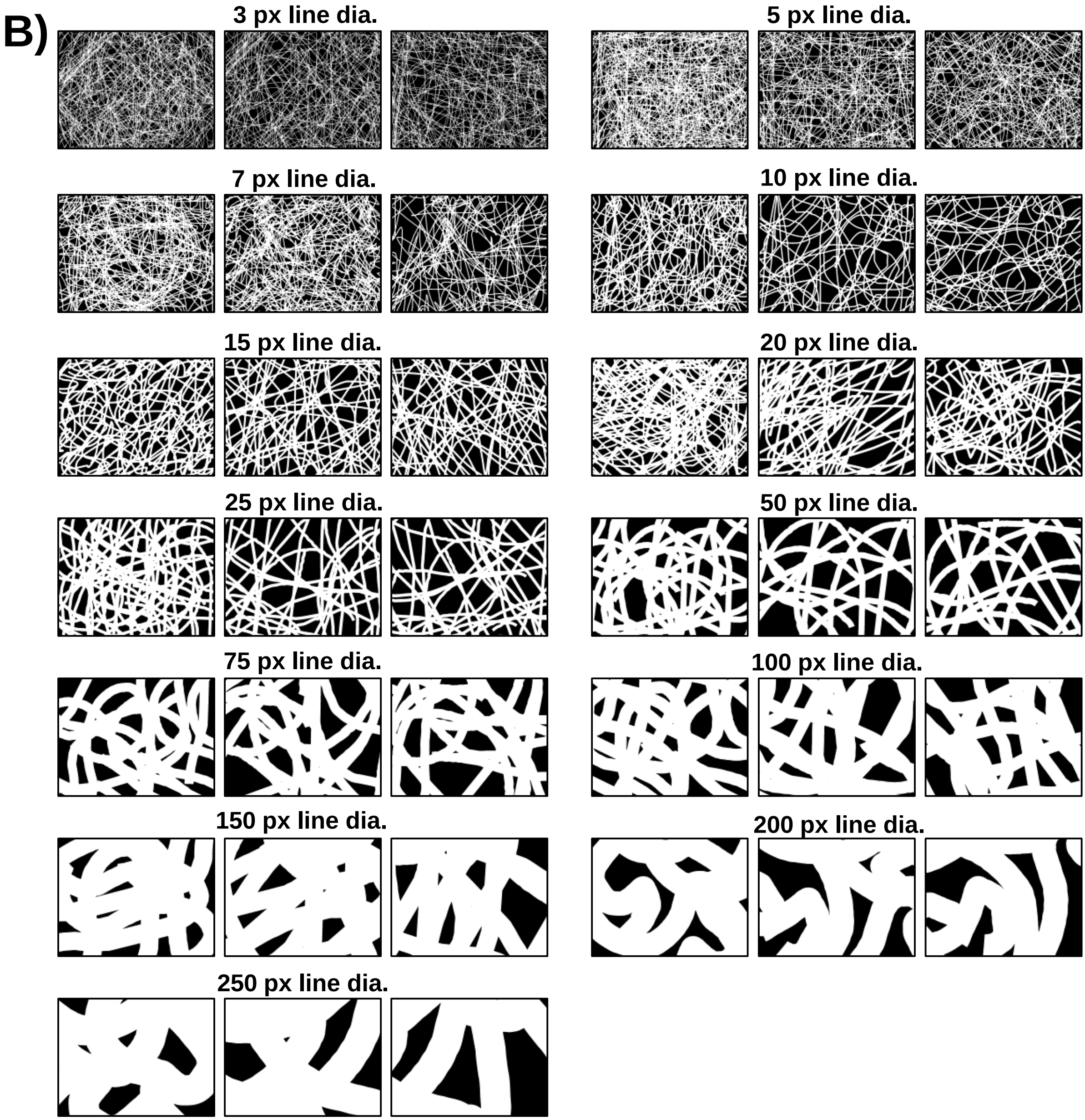

Figure S1B: Calibration Images Disordered, 1 Diameter (Disordered-1D)

C)

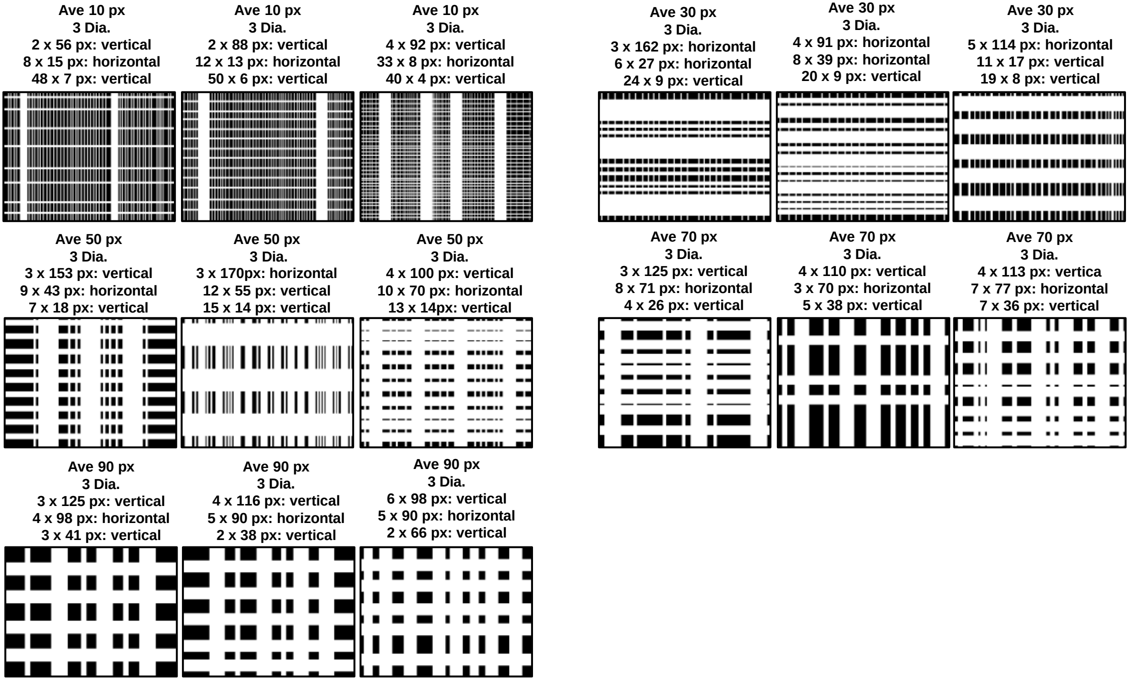

D)

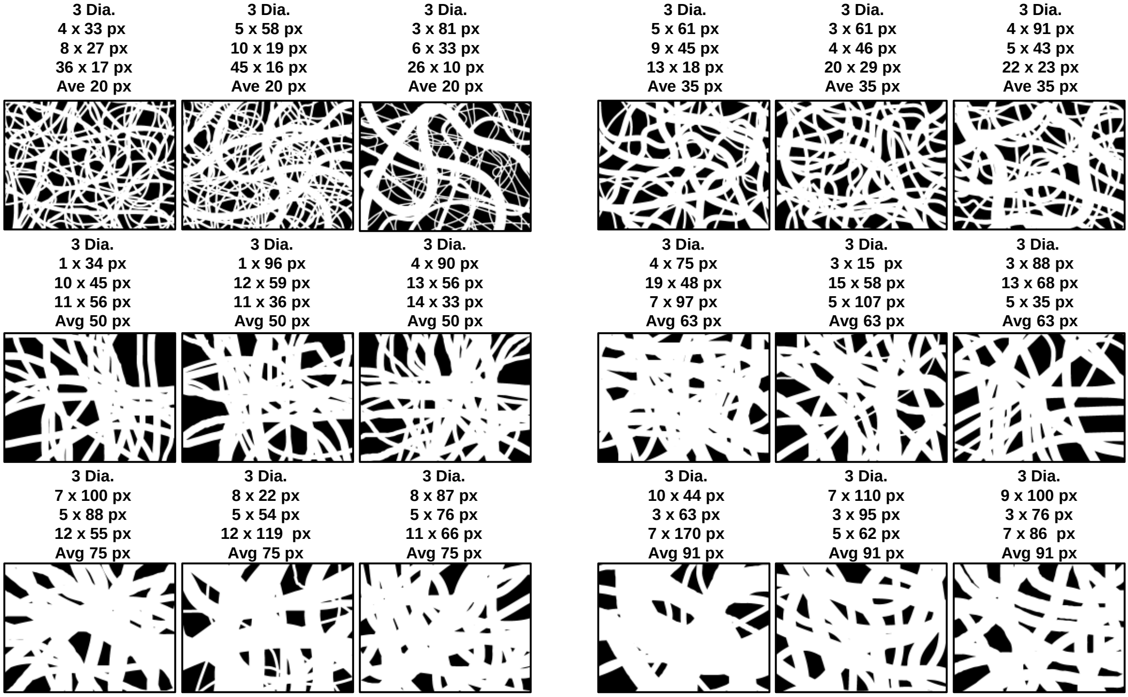

Figure S1: C) Calibration Images Ordered, 3 Diameter (Ordered-3D). The images within each set of three have the same average fiber diameter. This set is designed to validate DiameterJ Super Pixel's and Histogram's ability to determine a 'mean global fiber dia.' Images in each set of three are analyzed together and the 'errors' for the 'mean global fiber dia.' are averaged. D) Calibration Images Disordered, 3 Diameter (Disordered-3D). The images within each set of three have the same 'mean global fiber dia.' This set is designed to validate DiameterJ Super Pixel's and Histogram's ability to determine a 'mean global fiber dia.' Images in each set of three are analyzed together and the 'errors' for the 'mean global fiber dia.' are averaged. This fibers are dis-ordered in this set of images to make them better mimics of real electrospun nanofiber SEM images.

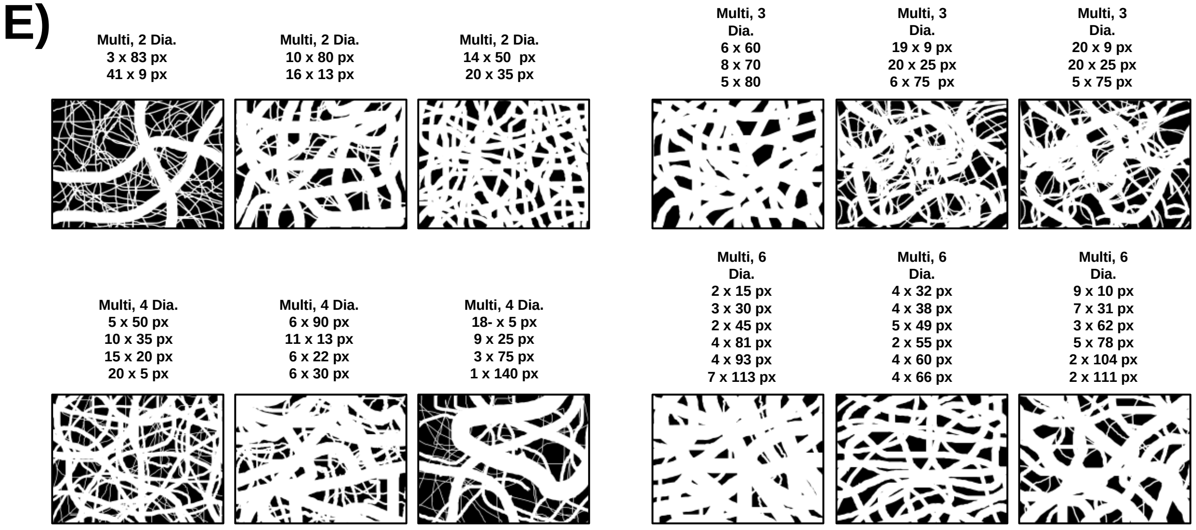

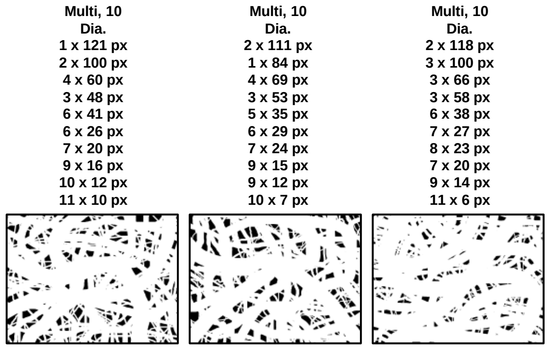

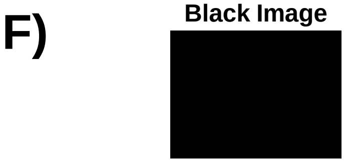

Figure S1: E) Calibration Images Multi-Diameter, Disordered (Multi-Dia.) The images within each set of three have different average fiber diameters. This set is designed to validate DiameterJ Histogram's ability to distinguish between multiple fiber diameters via multi-modal peak-fitting of histograms. Images in each set of three are analyzed individually and the 'errors' for each fiber dia. in each image are averaged. Thus, a 'mean global fiber dia.' is not provided for each image below. Data from the 10-line images were not included in the paper since the data were not interpretable. F) This image was used to validate that DiameterJ would not produce any fiber diameters measurements if no fibers were present in an image.
